# Magnetic resonance imaging measurement of the femoral antetorsional angle: Comparison with computer tomography and clinical application in a diagnostic algorithm

**DOI:** 10.1002/jeo2.70293

**Published:** 2025-06-05

**Authors:** Ewa Tramś, Maciej Liszka, Krzysztof Kuliński, Kamila Malesa, Marcin Tyrakowski, Jerzy Białecki, Rafał Kamiński

**Affiliations:** ^1^ Department of Musculoskeletal Trauma and Orthopaedics, Centre of Postgraduate Medical Education Gruca Orthopaedic and Trauma Teaching Hospital Otwock Poland; ^2^ Department of Orthopedics, Centre of Postgraduate Medical Education Gruca Orthopaedic and Trauma Teaching Hospital Otwock Poland; ^3^ Department of Spine Disorders and Orthopaedics, Centre of Postgraduate Medical Education Gruca Orthopaedic and Trauma Teaching Hospital Otwock Poland

**Keywords:** computed tomography, knee, magnetic resonance, paediatric sports medicine, patella

## Abstract

**Purpose:**

The purpose of this study was to compare five different femoral torsion measurement methods using computed tomography (CT) and magnetic resonance imaging (MRI), and to develop an MRI‐based rotational profiling technique that is comparable to and as repeatable as CT.

**Study Design: Diagnostic Study Methods:**

This prospective study evaluates patients who underwent lower limb rotational profile assessment by MRI and CT between June 2022 and October 2023. Five different measurement methods (Reikerås, Lee, Tomczak, Murphy and Jarrett) were compared to determine the best agreement. The Reikerås method was selected and further evaluated for intraobserver and interobserver reliability, as well as the learning curve, using intraclass correlation coefficients (ICCs). Reliability was assessed using ICCs, while agreement was evaluated through Bland–Altman analysis. Additionally, receiver operating characteristic (ROC) curve analysis with the area under the curve (AUC) was performed.

**Results:**

ICCs for the agreement between MRI and CT measurements indicated strong reliability (0.904 ± 0.022). The ICCs for intraobserver and interobserver reliability were 0.887 ± 0.026 and 0.871 ± 0.0471, respectively. Bland–Altman plots revealed a mean difference of −0.98° with limits of agreement ranging from −7.89° to 5.93°. The optimal cut‐off point for CT referral was calculated based on ROC curve analysis of MR‐based femoral torsion assessment, identified using the Youden index, at 19.67°, with an AUC of 0.998 ± 0.003, indicating excellent diagnostic accuracy. The learning curve showed an optimal ICC > 0.966 after 63 readings.

**Conclusion:**

The Reikerås MRI measurement method could replace CT, especially in the paediatric population, as it has shown high agreement, comparable reliability and reproducibility. The cut‐off point for this method is 19.67°.

**Level of Evidence:**

Level II.

**Abbreviations**
AUC: area under the curve
CI: confidence interval
ICC: intraclass correlation coefficient
ROC: receiver operating characteristic
TT‐TG: tibial tuberosity‐trochlear groove

## INTRODUCTION

Patellofemoral instability primarily affects female adolescents in their second decade of life, with incidence rates ranging from 5.8 to 29 per 100,000 patients [8, 9]. It encompasses complex disorders involving bone abnormalities and soft tissue restraints, which, in some cases, can lead to the development of patellofemoral arthritis. The underlying causes of patella instability are multifactorial, including both, soft tissue and osseous abnormalities [2, 19]. Surgical procedures should follow the principles of Lyon's school ‘menu à la carte’, which addresses all identified issues. Every patient should undergo a series of imaging studies, including: strict lateral radiographs, magnetic resonance imaging (MRI) to assess soft tissue, trochlear dysplasia, patella alta and the tibial tubercle‐trochlear groove distance, computed tomography (CT) for identifying rotational abnormalities and aiding in surgical planning [8, 9, 10, 12]. Recently, MRI has become the gold standard for diagnosing patellofemoral disorders, and identifying pathological anatomy, superseding CT scans. Knee MRI is highly effective for evaluating and planning surgery in cases of patellofemoral pain syndrome, as well as in both initial and recurrent patellar dislocations [8, 28, 39]. However, CT remains the gold standard for the quantitative measurement of femoral torsion [8, 33, 36].

In recent years, researchers have advocated for the use of MRI instead of CT to diagnose torsion abnormalities associated with patellar dislocation [3, 11, 13, 31, 35]. A positive correlation between radiation dose from CT scans and increased cancer risk is well‐documented [27, 38]. The radiation dose from a standard lower limb rotational profile scan is approximately 0.46–0.49 mSv. Paediatric torsion measurement can be performed using an ultra‐low‐dose protocol (range: 0.14–0.5 mSv). However, MRI remains the recommended radiation‐free method [18, 25, 37, 38]. Given concerns about radiation exposure, particularly in adolescents and children, there is a strong argument for replacing CT scans with radiation‐free techniques, such as MRI. Furthermore, using a single diagnostic modality (MRI) instead of two (MRI and CT) could reduce the financial burden on the healthcare system in most cases. Although distal femoral measurement is well established as the posterior condylar line, the definition of the proximal line remains in consensus [40].

The objective of this study was to compare femoral torsion measurements performed using CT versus MRI and to develop an MRI protocol with hip scans that are comparable, easy to learn, and repeatable, regardless of the assessor's experience. A secondary aim was to evaluate the diagnostic accuracy of MRI in femoral torsion measurement using receiver operating characteristic (ROC) and AUC metrics and to assess both interobserver and intraobserver reliability. The hypothesis was that this study would help establish a cut‐off point for CT referrals in femoral torsion assessment, thereby reducing the number of CT scans.

This study provides novel insight into torsion measurements on MRI and introduces a new analytical approach to propagate using MRI instead of CT. By leveraging comparative analysis of five different techniques, we aim to improve the diagnostic process for patella dislocation. Our research offers a comprehensive reliability analysis across both imaging techniques. This dual‐modality comparison can guide clinicians in selecting the most consistent imaging tool for diagnosis and follow‐up.

## MATERIALS AND METHODS

### Participants

**Figure 1:**
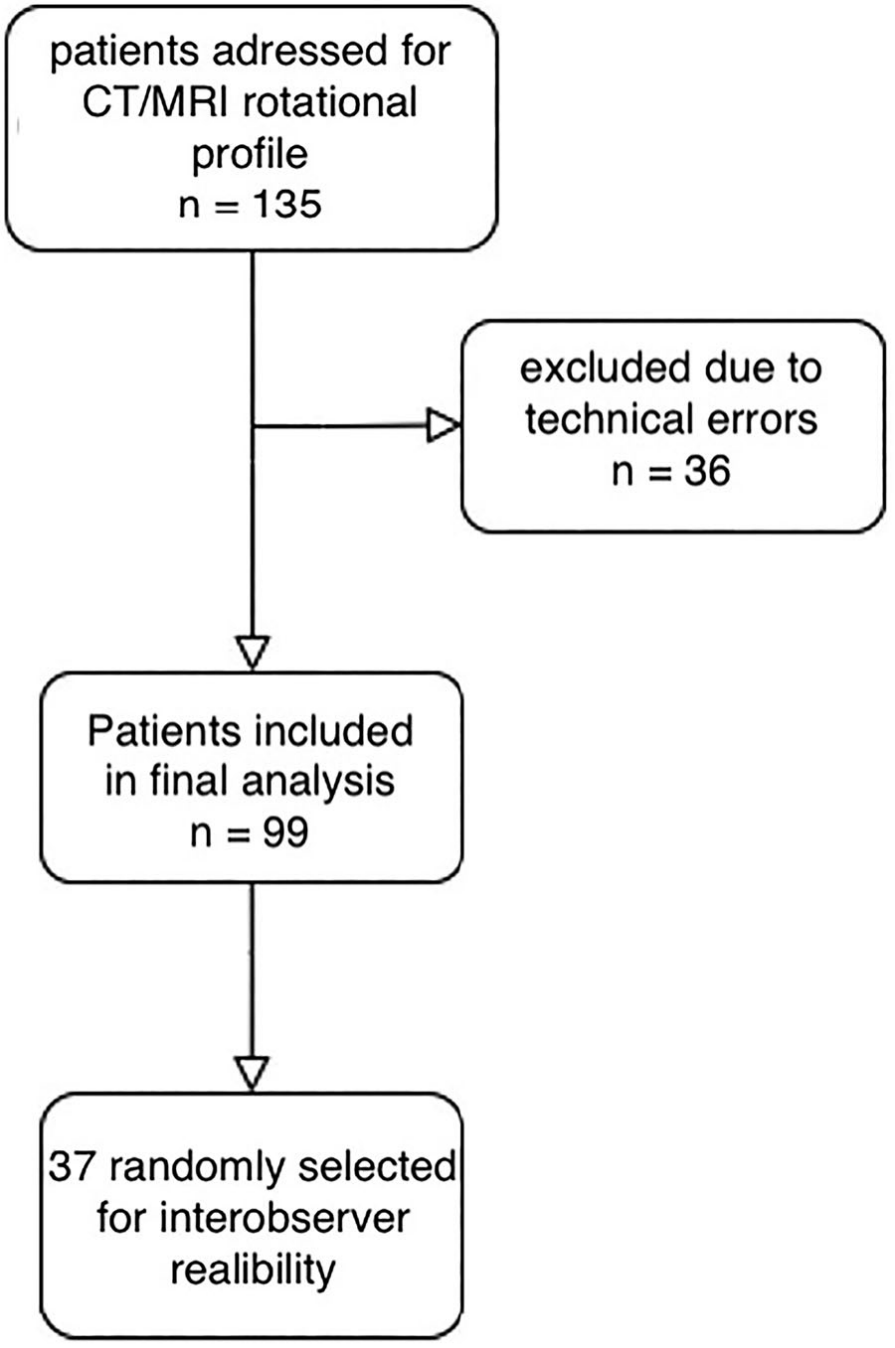
Flowchart of participant enrolment. CT, computed tomography; MRI, magnetic resonance imaging.

This prospective study was conducted following the approval of the Ethics Committee (approval number 12/2022) at a single orthopaedic academic centre and in accordance with the STARD checklist. Between June 2022 and October 2023, 135 consecutive participants scheduled for knee surgery were considered for inclusion (Table 1, Figure 1). The inclusion and exclusion criteria are detailed in Table 2. These participants underwent standard CT imaging of the hip, knee, and ankle for rotational assessment, as well as MRI of the knee with axial scans of the proximal femur. The first 20 patients were qualified as the test sample and excluded from the study. Another 16 were excluded due to technical errors while undergoing MRI, resulting in a final cohort of 99 patients. The examination and qualification of participants for surgery was performed by a senior orthopaedic surgeon, with eligibility set as a minimum of two incidents of patella dislocation for participants with patella dislocation or knee pain due to cartilage lesions. To ensure sufficient power for ROC‐AUC analysis, the number of participants enroled in the study was based on the frequency of participants with femoral torsion above 25° in the departmental database. The estimated sample size was 99 participants, assuming a 10% dropout rate.

**Table 1:** Demographic characteristics.

Age (years)	21–87 (mean 60.83 ± 16.97)
Sex (female–male)	54 (54.5%) to 45 (45.5%)
Knee osteoarthritis	78 (78.8%)
Patella dislocation	21 (21.2%)

**Table 2:** Inclusion and exclusion criteria.

Inclusion criteria	Exclusion criteria
18–99 years	Technical errors
Scheduled surgery (patella surgery or total knee replacement)	Previous bone surgeries on lower extremity
No previous bone surgery on lower extremity	
Minimum two patella dislocation incidences or clinical osteoarthritis	

### Imaging protocol

#### MRI determination of torsion technique

For MRI imaging, participants were positioned supine on the scanner. Three plane localizers of the hip, knee and ankle were taken before the procedure. In addition to standard knee MRI sequences, four additional MRI sequences were performed (20 transverse and 6 coronal slices through hips and ankles were added for measuring femoral torsion). T‐1 proton density‐weighted turbo spin echo hip transverse sequences were performed in the following parameters: repetition time 1020.00//32.00, echo train length 8, matrix 532 × 896, section thickness 4 mm, flip angle 140, acquisition time 3 min. Subsequently, T‐1 coronal localizer of the hip was performed in the following parameters: repetition time 1850.00//30.00, echo train length 17, matrix 220 × 320, section thickness 5 mm. For knee T‐2 proton density‐weighted turbo spin echo transverse sequences with fat suppression were performed with the following parameters: repetition time 2100.00//33.00, echo train length 9, matrix 320 × 320, section thickness 4 mm, flip angle 130, acquisition time 2 min 47 s. Transverse knee sequences were compared to transverse knee localizer, and every case which was rotated inconsistently according to localizer was excluded from the study. The addition of an extra hip sequence takes an average of 3 min.

#### CT determination of torsion technique

For CT imaging, participants were in positions identical to the MRI examination: supine with feet first into the scanner, knees extended side by side, toes pointing up. A hip scan was performed first, and a second scan covered the knee. CT scans were performed using a Toshiba Aquilion ONE scanner in the following parameters: FFS position; Toshiba Aquilion ONE; 120 kV, 225 mAs; SC: 500.00 mm; AW9; GW 5 mm with an average radiation dose of 1.62 mSv.

### Image analysis

**Figure 2:**
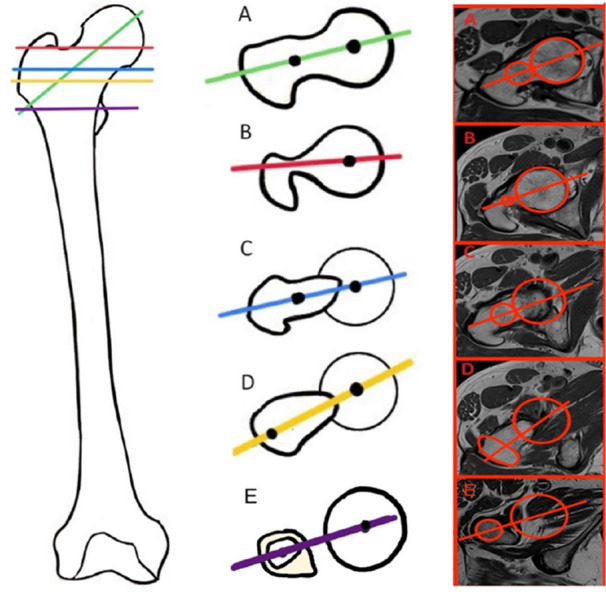
Define proximal reference line on proximal femur for anatomic landmarks selection on axial images. A. Jarrett, B. Lee, C. Reikerås, D. Tomczak and S. Murphy.

Analysis of CT and MRI examinations was performed by four researchers, three of whom had at least 10 years of experience in MRI and CT measurements. Prior to final measurements, 20 MRIs and their corresponding CT scans were assessed to establish the measurement method with the best agreement (Table 3). Five commonly used methods for measuring the femoral neck version were evaluated: Reikerås, Lee, Tomczak, Murphy and Jarrett (Figure 2) [16, 22, 26, 30, 35].

**Table 3:** Summarizing table five commonly used measurement methods.

	CT (mean ± SD)	MRI (mean ± SD)
Reikerås	9.05 ± 6.607	9.12 ± 6.861
Lee	6.38 ± 8.592	7.6 ± 7.351
Tomczak	19.13 ± 8.068	17.8 ± 8.124
Murphy	17.16 ± 9.337	12.39 ± 9.387
Jarret	9.04 ± 6.674	9.13 ± 6.908

Abbreviations: CT, computed tomography; MRI, magnetic resonance imaging; SD, standard deviation.

**Figure 3:**
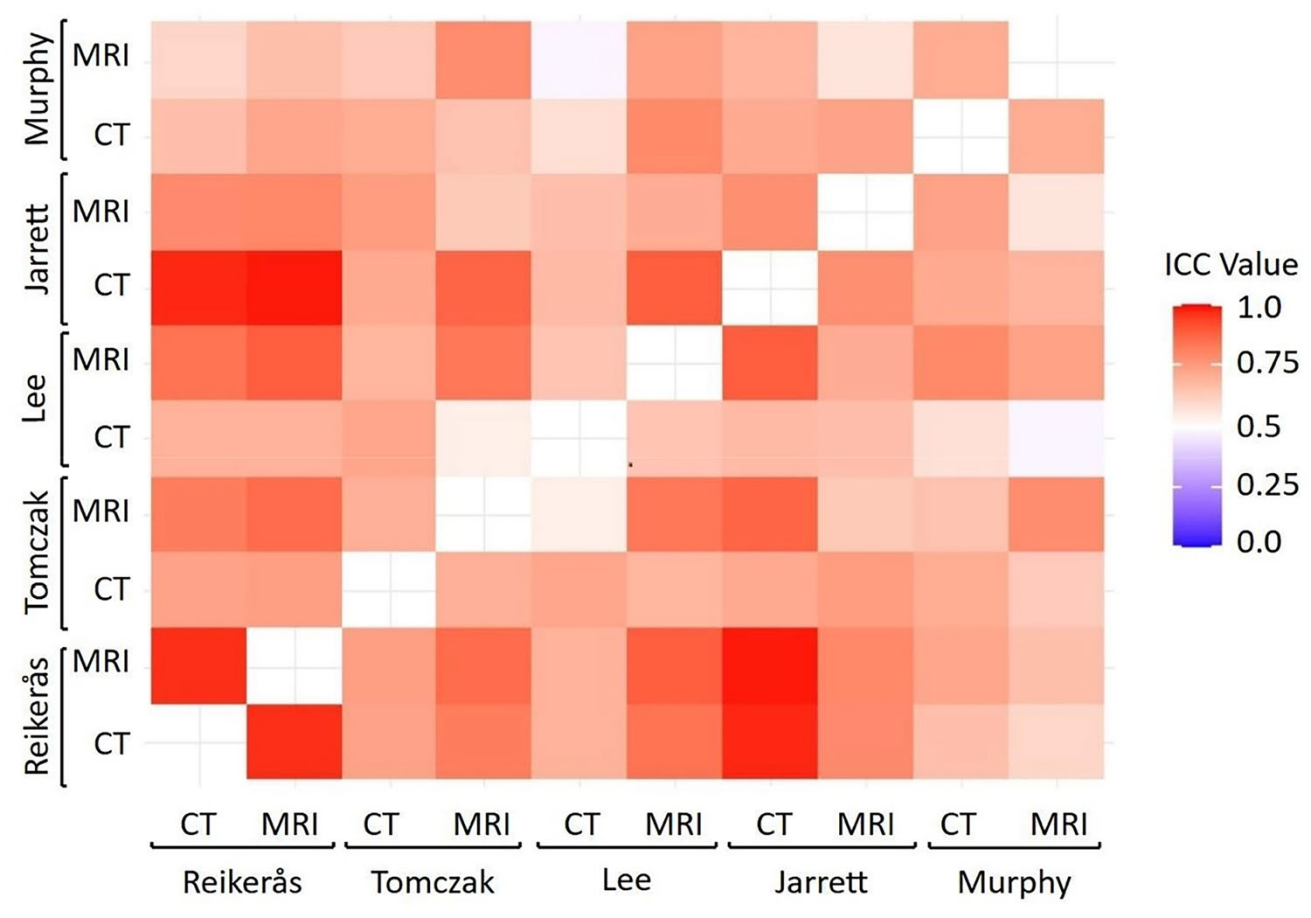
The consistency heatmap compares all four MRI and CT measurement methods (Jarrett, Lee, Reikerås, Tomczak and Murphy). CT, computed tomography; ICC, intraclass correlation coefficient; MRI, magnetic resonance imaging.

The intraclass correlation coefficient (ICC) analysis demonstrated varying degrees of agreement and consistency between MRI‐ and CT‐based femoral torsion measurements across the evaluated methods. The Reikerås method exhibited excellent agreement (ICC = 0.969; 95% CI: 0.924–0.988), strongly supporting MRI as a viable alternative to CT for this measurement approach. The Jarrett method showed good agreement (ICC = 0.761; 95% CI: 0.482–0.899), while the Tomczak (ICC = 0.711; 95% CI: 0.402–0.875), Lee (ICC = 0.647; 95% CI: 0.307–0.842) and Murphy (ICC = 0.637; 95% CI: 0.206–0.848) methods demonstrated moderate agreement (Figure 3).

**Figure 4:**
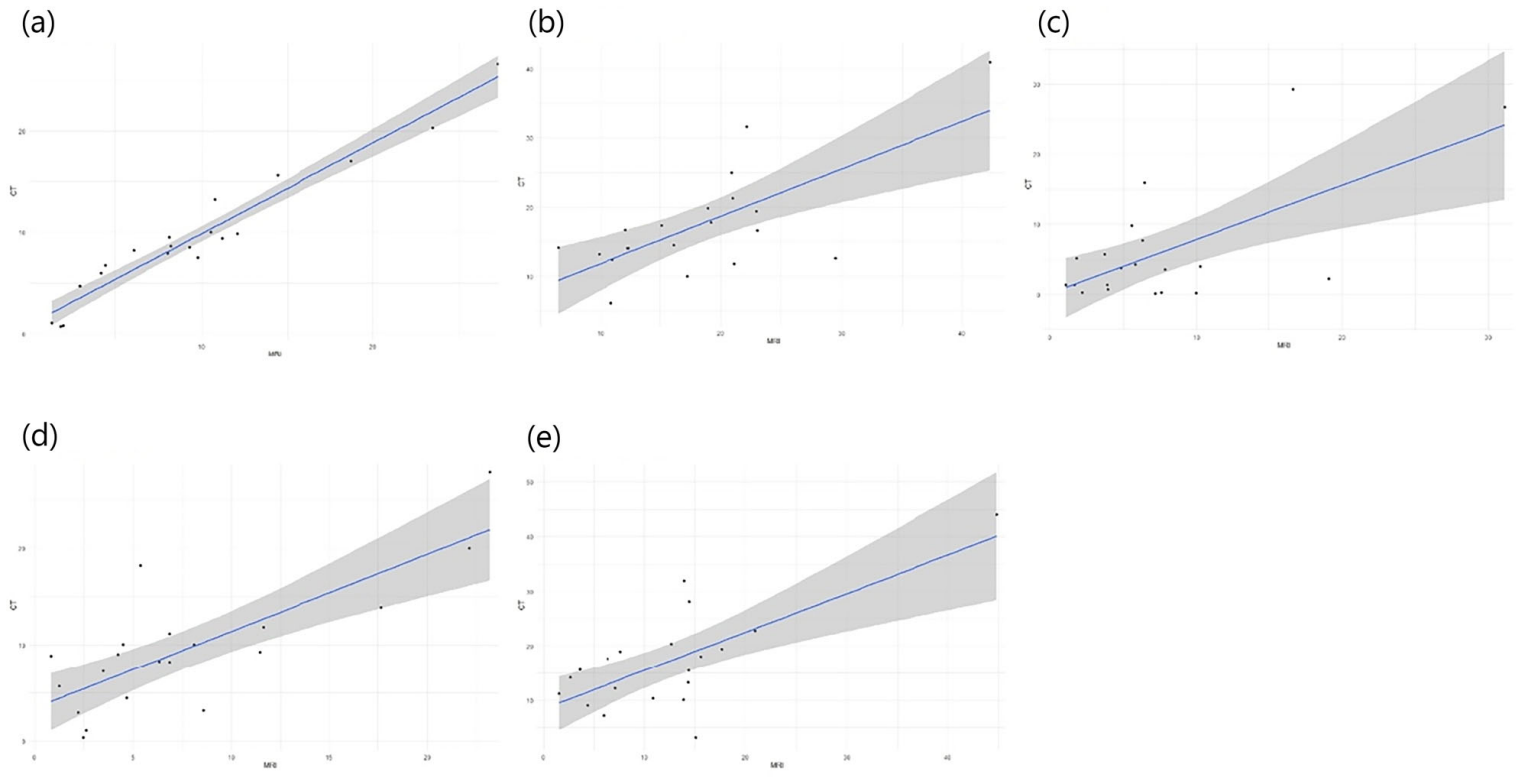
Linear regression. (a) Reikerås, (b) Tomczak, (c) Lee, (d) Jarrett and (e) Murphy.

Linear regression analyses revealed statistically significant correlations between MRI and CT for all methods evaluated, with varying strengths. The Reikerås method exhibited the strongest linear relationship (*R* ^2^ = 0.942, *p* < 0.001), indicating that MRI results closely predict CT outcomes. Other methods showed moderate correlations: Jarrett (*R* ^2^ = 0.614, *p* < 0.001), Murphy (*R* ^2^ = 0.505, *p* < 0.001), Tomczak (*R* ^2^ = 0.496, *p* < 0.001) and Lee (*R* ^2^ = 0.433, *p* = 0.002) (Figure 4).

### Statistical analysis

Interobserver and intraobserver reliability were evaluated using ICCs. Agreement was assessed with Bland–Altman analysis, and the Fisher test was used to compare variances of the samples. The analysis was performed on 99 samples for agreement assessment and the generation of an ROC curve. For intraobserver reliability, the main researcher measured two times, randomised and blinded, for the first measurement of 99 samples after 3 months. For interobserver reliability, 37 randomized samples were analyzed by all four researchers, and subgroup comparisons were made between the primary researcher and each of the other researchers. ROC analysis was performed to calculate the AUC [41]. Reported sensitivity and specificity were used to estimate diagnostic parameters, to compare the discrimination ability of the described method, and to determine the optimal cut‐off points of the indices based on the Youden index [23]. Qualitative statistical analysis and network meta‐analysis were performed using R™ software 4.3.1 (R Foundation for Statistical Computing) [29]. The ICC analysis was performed using the irr package version 0.84.1 with bootstrap functions (boot package version 1.3‐28.1) for ICC SE and 95% CI assessment. The ROC‐AUC analysis was performed with pROC package version 1.18.4. Data for visualization were transformed using the reshape2 package (version 1.4.4). Graphs were created using the ggplot2 package (version 3.5.1). *p* values < 0.05 were considered to indicate statistical significance.

## RESULTS

### Patients' characteristics

The final study sample included 99 participants. The mean age was 60 years, with a range of 21–87 years. The population comprised 54 female and 45 male subjects. Patella dislocation was diagnosed in 21 participants, while osteoarthritis was diagnosed in 78 participants. The average torsion measurement was 10.75 ± 0.891° (95% CI: 8.996–12.495) using the MRI‐based technique and 11.9 ± 0.824° (95% CI: 10.289–13.521) using the CT‐based technique, both assessed according to the Reikerås method.

### Interobserver and Intraobserver reliability

**Figure 5:**
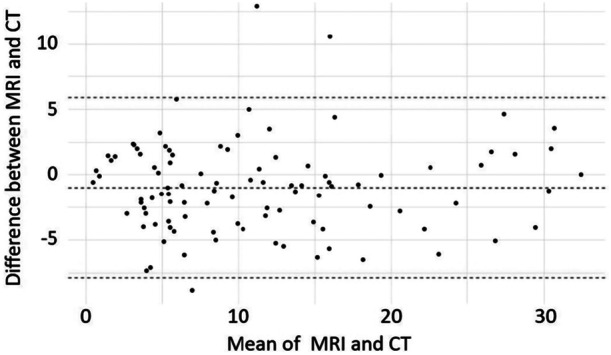
Bland–Altman plots assessing the agreement between MRI and CT measurements (Reikerås). CT, computed tomography; MRI, magnetic resonance imaging.

Interobserver and intraobserver reliability were good. ICCs for the agreement between MRI and CT measurements indicated high reliability (0.904 ± 0.022; 95% CI: 0.856–0.936; *p* = 5.73 × 10^−31^). Bland–Altman plots depicted the agreement between MRI and CT measurements (Figure 5), revealing a mean difference between the two of 0.98° with limits of agreement of 5.93 and −7.89, for upper (ULA) and lower (LLA) limit, respectively, for whole data set and mean difference of −0.37° (3.68, −4.42; ULA and LLA respectively) for last 37 measurements series. Intraobserver ICC was 0.887 ± 0.026 (95% CI: 0.837–0.923; *p* = 5.37 × 10^−35^) for all 99 measurements, while interobserver ICC was 0.871 ± 0.0471 (95% CI: 0.8–0.925; p = 3.99 × 10^−40^) for 37 measurements. Agreement levels among the researchers are presented in Table 4. The ICC values for intraobserver reliability (0.887 ± 0.026 for 99 measurements) and interobserver reliability (0.871 ± 0.0471 for 37 measurements) are clearly distinguished. This clarification specifies which ICC values pertain to the repeatability of measurements by the same operator and which relate to those between different operators.

**Table 4:** Intraobserver and interobserver correlation for each measurement technique.

	ICC	SE for ICC	*p* value	95% CI
Intraobserver	0.887	0.0263	5.37^−35^	0.837–0.923
Interobserver all 4	0.871	0.0472	3.99^−40^	0.800–0.925
Interobserver 1 vs. 2	0.83	0.0709	7.383^−11^	0.694–0.908
Interobserver 1 vs. 3	0.816	0.074	2.939^−10^	0.671–0.901
Interobserver 1 vs. 4	0.859	0.055	3.551^−12^	0.743–0.925

Abbreviations: CI, confidence interval; ICC, intraclass correlation coefficient.

### Diagnostic parameters

**Figure 6:**
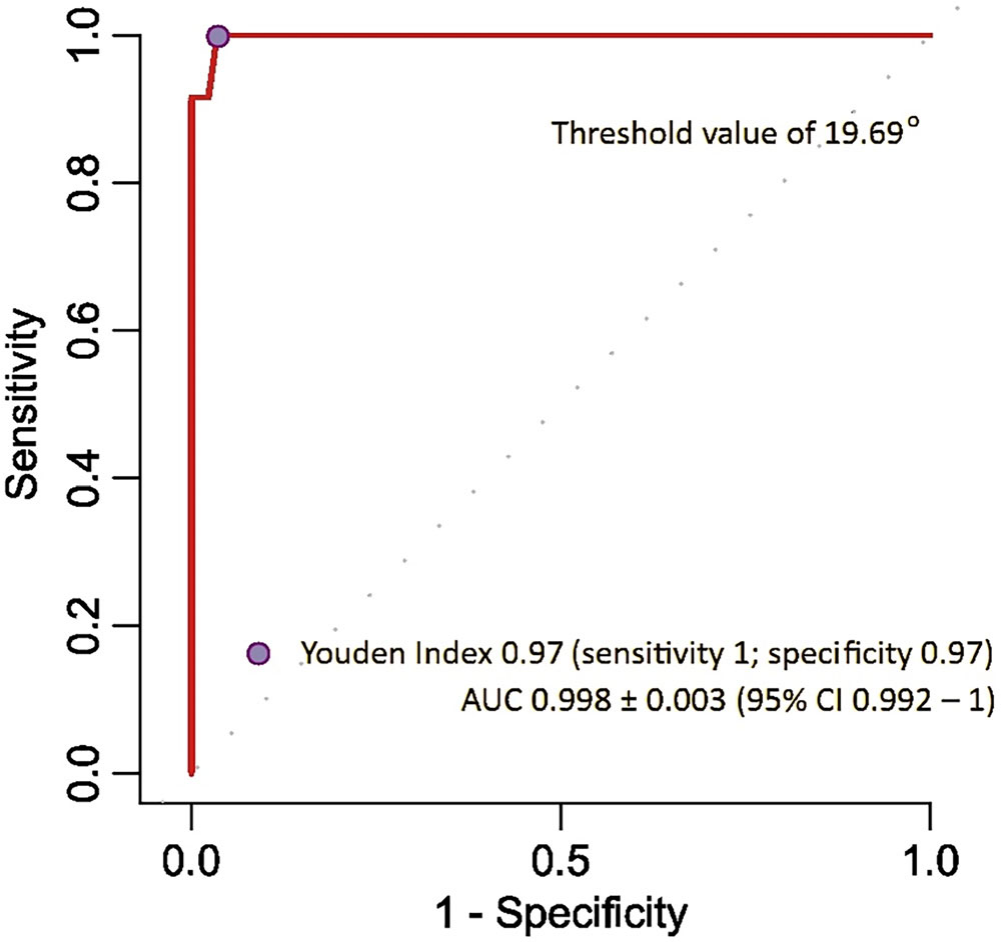
Receiver operating characteristic curve (area under the curve [AUC ± SEM]: 0.998 ± 0.003 [95% CI: 0.992–1]). Identification of the optimal cut‐off point for the MR‐measured angle of torsion referenced to CT scanning (Youden index: 19.69° [sensitivity: 1; specificity: 0.97]). CI, confidence interval; CT, computed tomography; MR, magnetic resonance; SEM, standard error of the mean.

To determine the optimal cut‐off point for the MR‐measured femoral torsion angle and to evaluate the diagnostic efficacy of MR‐based torsion assessment, the ROC curve and corresponding AUC were calculated using a CT threshold of greater than 25° (Figure 6). The AUC was 0.998 ± 0.003 (95% CI: 0.992–1), indicating excellent diagnostic accuracy. The optimal cut‐off point for MR‐measured femoral torsion, serving as a referral threshold for the CT‐based method, was identified as 19.69° (sensitivity: 1; specificity: 0.97). Below this point, a standard CT rotational profile should be performed. The three methods employed, Youden's index, closest top‐left and cost‐effectiveness, yielded the same cut‐off point, serving as the threshold above which classical CT limb torsional assessment should be recommended as a reference method.

### Learning curve

**Figure 7:**
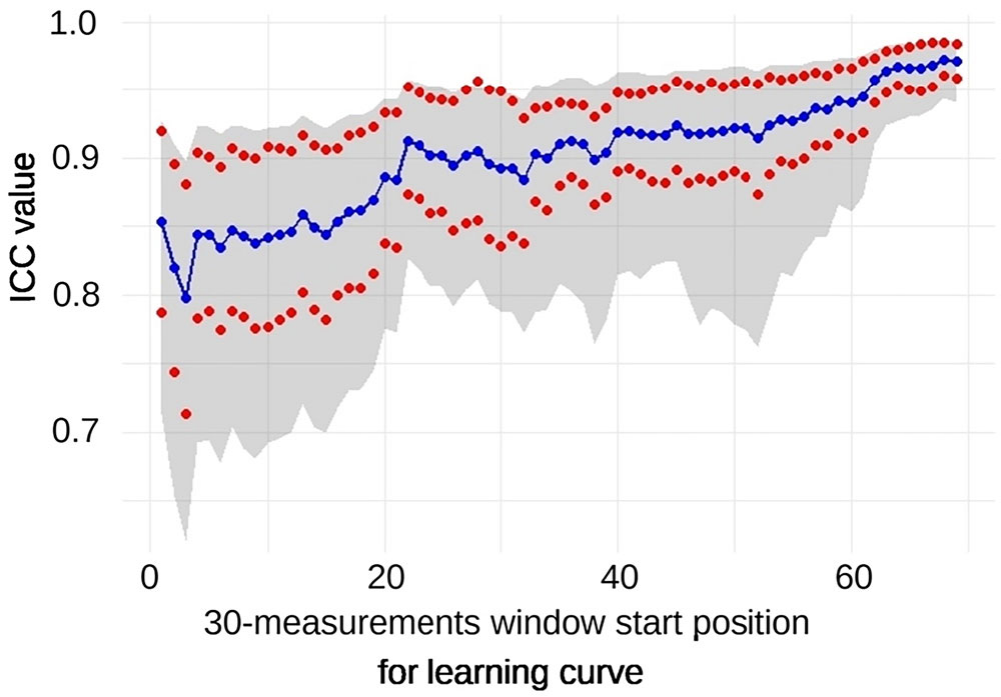
Learning curve for torsion measurements with a 30‐measurement window. Mean (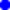) ± SEM (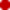), 95% CI (grey area).

The assessors' learning curve was calculated based on consecutive sets of 30 measurements. Samples were analyzed over time, showing an increase in ICC values with subsequent measurements. The initial agreement (ICC) for the first five series ranged from 0.797 ± 0.066 to 0.853 ± 0.083 (95% CI: 0.713–0.927; *p* = 1.99 × 10^−19^ and 95% CI: 0.620–0.898; *p* = 2.4 × 10^−8^, respectively), with progressive improvements to 0.966 ± 0.018 to 0.973 ± 0.012 (95% CI: 0.931–0.984; *p* = 1.99 × 10^−19^ and 95% CI: 0.944–0.987; *p* = 8.8 × 10^−21^, respectively). The learning curve reached an optimal ICC > 0.96 after 63 readings (Figure 7).

## DISCUSSION

CT is frequently employed by orthopaedic surgeons as the preferred imaging modality for evaluating the femoral torsion angle [4, 6, 24]. Despite some studies showing MRI to be comparable, and in certain aspects, such as the absence of radiation exposure, a superior technique, CT remains the gold standard for measuring femoral torsion [8]. This study demonstrated that MRI can serve as an alternative to CT, identified the most reliable and repeatable measurement method (Reikerås), and showed improvements over the limitations identified in previous studies.

The topic of x‐ray radiation exposure during CT has been a subject of discussion. Some researchers report that the relative risk of cancer development following CT scan utilization is estimated to be 5–17 times greater than with traditional x‐ray imaging [38]. Additionally, when assessing femoral torsion using CT, significant differences in measurements have been reported depending on the chosen CT‐measurement technique [17, 32]. Given the information above, and considering that a significant number of patients undergoing orthopaedic surgeries frequently undergo MRI, it is logical to employ this imaging technique for assessing femoral torsion. Various studies have examined different measurement methods [12, 15, 32, 35, 39]. Alter et al. conducted comparisons between axial‐oblique sequences and conventional axial slices, detecting more suitable measurement consistency at multiple time points for axial‐oblique femoral torsion assessments. They also found that decreased femoral torsion values were obtained with oblique‐axial compared to standard axial techniques. However, they did not compare MRI with CT images and only retrospectively included patients undergoing hip arthroscopy for femoroacetabular impingement (FAI) syndrome [1]. In their comparative analysis of femoral torsion measurements obtained from axial CT scans versus axial oblique MRI scans, Tomczak et al. suggested that the discrepancies observed in measurements can be attributed to the differing image orientations utilized in CT and MRI techniques [35]. Sutter et al. arrived at similar findings, demonstrating that measurements derived from axial oblique images do not accurately reflect true anatomical dimensions [34]. Botser et al. also observed the impact of patient positioning during MRI examinations on the measurement outcomes of femoral torsion. CT was found to have higher interobserver reliability and was larger than MRI, with a mean difference of 8.9. They concluded that MRI should not supplant CT in assessing this parameter. However, this high value of systemic bias may influence final results [5].

Contrary to the studies mentioned above, there are publications indicating comparable outcomes in evaluating femoral torsion with MRI and CT. Hesham et al. demonstrated that employing axial images of the pelvis and knee in MRI for adolescents facilitates reliable measurement of femoral torsion [15]. Schmaranzer et al. demonstrated that MRI produces results comparable to CT in the measurement of femoral torsion [31]. In this study, we made a similar observation. The optimal sequence that minimized these discrepancies was hip‐knee‐ankle, using short‐segment scans that specifically allow for the assessment of torsional changes in the limb, followed by a complete knee examination.

Quantifying femoral torsion accurately is critical for diagnosing and surgically managing torsional deformities. Both CT and MRI modalities offer multiple techniques for measuring this angle, each relying on distinct anatomical landmarks. Schmaranzer et al. demonstrated that femoral torsion values can vary substantially depending on the measurement location (proximal vs. distal) and the specific method used, with discrepancies up to 20°, especially in patients with excessive torsion [32].

This study addresses a significant gap: whether MRI‐based torsion measurements are as susceptible to method‐related variability as CT‐based measurements, and whether a standardized MRI protocol can reliably replace CT in clinical practice. We comprehensively evaluated five femoral torsion measurement techniques (Reikerås, Lee, Tomczak, Murphy and Jarrett) using both CT and MRI. Each method employs a slightly different anatomical reference line—some more proximal or distal than others. Careful selection of measurement method—ideally one with validated reliability and high inter‐modality agreement—is critical if MRI is to serve as a viable diagnostic alternative to CT. Of particular note, the Reikerås method provides a reproducible and accurate framework for future clinical and research applications.

This study offers several strengths. First, research is prospective and compares MRI to CT measurements using the most comparable and repeatable method (Reikerås) chosen from five distinct methods. Additionally, standard axial sequences were compared with intertrochanteric sequences. To minimize the bias from potential movements of the patient's leg, initially rapid scanning of the hip and knee was conducted for localization and subsequently compared the final sequences with the localizer. The results were validated through both intraobserver and interobserver reliability assessments. The participants span various age groups and, unlike in many earlier studies, do not present with hip deformities (such as FAI, hip dysplasia, coxa valga or Perthes‐like deformity). Patients with specific torsional angle abnormalities (e.g., patellar dislocation) alongside a standard population (e.g., individuals with osteoarthritis of the knee) were considered.

There are several limitations. Patients with hip deformities were not included and only adults were considered, with an average age of 60 years, which may seem too old given that our target group is adolescents and young adults (20–30 years old). To confirm that this method of measurement is the most reliable for assessing torsion in patellofemoral disorders, another prospective study focusing on a younger population is necessary. Additionally, while there were employed patients with arthrosis or patellofemoral disorders, there is a need for further research, predominantly involving adolescent patients with patellofemoral disorders.

It is also worthwhile to consider other parameters that can be assessed using the previously mentioned diagnostic methods—one of which is the tibial tuberosity‐trochlear groove (TT‐TG) distance. This parameter is pivotal for evaluating patellofemoral instability. Prior studies have demonstrated substantial differences in TT‐TG measurements when comparing MRI and CT [7, 20]. Such findings should be taken into account during surgical planning if MRI is used, given that MRI may underestimate the TT‐TG distance compared with CT. This serves as an important example of why individual measurement methodologies must be carefully adapted to the imaging technique before making critical clinical decisions.

Another factor to consider when comparing MRI to CT is the balance between availability, scan duration, and cost. Although CT may appear more accessible and cost‐effective, many patients already require MRI to assess other knee structures, making an additional CT potentially unnecessary and thus reducing overall diagnostic costs. While it is true that CT scans are faster, extending the MRI to include the relevant anatomy—rather than scheduling a separate CT—can also result in both time and cost savings [14, 21]. Overall, these factors need to be evaluated comprehensively to draw clear conclusions.

In conclusion, both MRI and CT scans have proven equal value in the accurate assessment of femoral torsion. Therefore, given the absence of radiation risk associated with MRI, we suggest that MRI be considered the primary diagnostic modality for patients suspected of having torsional abnormalities of the femur. CT should be reserved as a confirmatory technique when MRI‐based assessment of femoral torsion exceeds 19.69°, particularly in cases requiring surgical planning. This approach not only maximizes diagnostic efficacy but also prioritizes patient safety by minimizing unnecessary radiation exposure.

## AUTHOR CONTRIBUTIONS

**Rafał Kamiński**: Conceptualization; methodology; formal analysis; investigation; writing—review and editing; supervision. **Ewa Tramś**: Methodology; writing—original draft preparation. **Kamila Malesa**: Writing—original draft preparation. **Maciej Liszka**: Resources. **Krzysztof Kuliński**: Resources. **Marcin Tyrakowski**: Supervision. **Jerzy Białecki**: Supervision.

## CONFLICT OF INTEREST STATEMENT

The authors declare no conflicts of interest.

## ETHICS STATEMENT

The study was approved by the Bioethics Committee of the Centre of Postgraduate Medical Education (Approval no. 12/2022). Informed consent was obtained from all individual participants included in the study.

## Data Availability

The data that support the findings of this study are available from the corresponding author upon reasonable request.
